# Nonlinear Dynamics and Applications

**DOI:** 10.3390/e27070688

**Published:** 2025-06-27

**Authors:** José M. Amigó, Fernando Montani

**Affiliations:** 1 Centro de Investigación Operativa, Universidad Miguel Hernández, 03202 Elche, Spain; 2 Instituto de Física de La Plata (IFLP), CONICET-UNLP, La Plata B1900, Buenos Aires, Argentina; f.montani@fisica.unlp.edu.ar

Nonlinear dynamics is the study of dynamical systems in finite dimensions, whether in discrete or continuous time, in which the evolution equation (a difference or differential equation, respectively) is not linear in the state variables [1,2]. A potential result of nonlinear dynamics is sensitivity to initial conditions (i.e., small perturbations of the initial condition can lead to large divergences in the corresponding trajectories in the state space over time), making such systems unpredictable on a given time scale [3]. Sensitivity to initial conditions, popularized as the butterfly effect, is a hallmark of deterministic chaos [4,5,6]. As the well-known example of the logistic parabolas shows, it suffices a quadratic term in the evolution equation for a (in this case, one-dimensional) system to exhibit, for suitably chosen parameters, a mind-wobbling complex behavior [7,8]. And low-dimensional chaos is just the entry point to an increasingly weird and fascinating world as the dimension increases [9,10].

Although nonlinear dynamics is as old as Newtonian dynamics (as its name reveals), it is generally acknowledged that the formulation of theoretical properties and qualitative methods started with the work of Poincaré on the three-body problem [11,12], and continued with Lyapunov [13], Birkhoff [14], Kolmogorov [15] and many other towering figures of pure and applied mathematics. The list of new, seminal concepts introduced in mathematics in the development of nonlinear dynamics is impressive: recurrence, homoclinic and heteroclinic orbits and tangles, invariant measures, ergodicity, Lyapunov exponents, metric and topological entropies, attractors, chaos, bifurcations, fractals, Julia sets, horseshoes, time-delay embedding, and many more [16,17]. In fact, the conceptual framework of nonlinear dynamics belongs to the core of modern mathematics. Furthermore, nonlinear dynamics has important intersections with other fundamental areas of mathematics such as ordinary differential equations, ergodic theory, statistics and probability theory.

Traditionally, real world applications of nonlinear dynamics have focused on the understanding of complex phenomena in nature. To mention a few representative examples, nonlinear dynamics is used in physics to study turbulence, weather evolution, nonlinear oscillations and chaotic synchronization [18,19,20]. In biology, we can find applications to population dynamics, epidemiology and heart dynamics [21,22,23]. And in engineering, it help in understanding mechanical vibrations, design control systems and electrical circuits [24,25]. More recently, the applications of nonlinear dynamics have also been extended to social sciences, e.g., economy and sociology, especially as data-driven analyses [26]. At the same time, new areas of research have emerged, such as chaotic cryptography [27], computational neuroscience [28] and nonlinear time series analysis [29]. In sum, in many complex phenomena amenable to the dynamical approach, nonlinear dynamics has enriched our understanding, bringing light to old and new problems alike.

Precisely, the research of Professor Osvaldo Rosso falls into nonlinear time series analysis, nonlinear dynamics, information theory, complex networks and their applications to physics, biology and medical sciences. In this regard, let us highlight his work on the differentiation between deterministic chaos and randomness [30], as well as the characterization of signals by means of several generally called entropy–complexity planes [31]. In doing so, he has also proven to be a driving force in the application of ordinal methods to the analysis of time series [32]. Professor Rosso was nominated among the top 5% of researchers with high impact in biological applications in the years 2022, 2023 and 2024.

In addition to the vast and influential work of Professor Rosso on the nonlinear and complexity sciences, we would also like to underline his leadership and mentoring, especially in South America, where he has nurtured a large and very active community of students and collaborators in the field of nonlinear time series analysis with a strong emphasis on applications. In recognition of his distinguished and productive scientific career, this Special Issue on “Nonlinear Dynamics and Applications” is dedicated to him on the occasion of his 70th birthday (15 October 2024).

This Special Issue comprises eighteen contributions, one of them being a review article and the rest being research articles. For the sake of this Preface, the research articles have been grouped into five topics: nonlinear time series analysis, dynamical systems and models, physics, chaos synchronization, and networks. In the following, we briefly describe all these articles.

Knowing the scientific trajectory of Professor Rosso, it is not surprising that a significant part of the contributions deal with time series analysis. Thus, Guisande et al. examine in [33] awake human intracranial electroencephalography (iEEG) data from normal brain regions to explore how biological sex influences these dynamics. The iEEG data were analyzed using permutation entropy and statistical complexity in the time domain and power spectrum calculations in the frequency domain. This study emphasizes the importance of considering sex as a biological variable in brain dynamics research, which is essential for improving the diagnosis and treatment of neurological and psychiatric disorders. Zunino et al. [34] quantify the similarity between two time series with the permutation Jensen–Shannon distance (JSD), i.e., the square root of the Jensen–Shannon divergence of the time series in the ordinal representation, with the probability distributions being estimated by the frequencies of the corresponding ordinal patterns. The authors show that the JSD is a powerful and robust metric for identifying the precise temporal scales at which two distinct time series exhibit ordinal similarity. Valverde et al. [35] study the differential behavior of the ECG during occlusion of both the left anterior descending (LAD) and right anterior coronary artery (RCA), respectively, in subjects with acute myocardial ischemia. To this end, the authors use ordinal patterns and locate the resulting signals in the causal complexity plane. The authors find that the locations for subjects with LAD ischemia, RCA ischemia and controls are different, making it possible to discriminate the three groups. A similar study is performed by Duarte et al. [36], this time to study sleep stages with generalized weighted permutation entropy (GWPE) and standard permutation entropy. Comparison of the results shows that GWPE significantly enhances sleep stage differentiation, which makes GWPE a valuable tool for the diagnosis of sleep disorders. The list of contributions to the topic of time series analysis continues with [37]. In this article, Olivares et al. show how ordinal patterns and metrics derived from them can be used to assess the effectiveness of detrending methods. As an interesting application, the authors apply their method to data representing the evolution of delays in major European and US airports, obtaining operational conclusions about how delays propagate in the two systems. Lange and Hauhs study the complexity of environmental time series in [38]. Specifically, the authors analyze selected long-term data from three headwater catchments in Lower Saxony (Germany). The metrics used, based on ordinal pattern statistics, comprise permutation entropy, permutation complexity and Fisher information, as well as their generalized versions (*q*-entropy and *α*-entropy). The analysis tools include Tarnopolski diagrams, ordinal pattern position slopes, horizontal visibility graphs and the exponent of the decay of the degree distribution. As a result, the authors characterize the dynamics of those systems. Finally, Suriano et al. [39] investigate the temporal evolution of cryptocurrency time series using information measures such as complexity, entropy, and Fisher information, with the objective of differentiating between various levels of randomness and chaos. Complexity–entropy causality plane (CECP) analysis reveals that daily cryptocurrency series with lengths of two years or less exhibit chaotic behavior, while those longer than two years display stochastic behavior.

The following contributions belong to the topic of dynamical systems and models. In the context of evolution forecasting of epidemic outbreaks from deterministic compartmental models, Rojas-Venegas et al. [40] extend the classical deterministic compartmental models to a quantum-like formalism to explore whether the uncertainty of epidemic forecasts is also shaped by the stochastic nature of epidemic processes. The authors conclude that the stochasticity of contagion and recovery processes poses a natural constraint for the uncertainty of epidemic forecasts. Quintanilha Valente et al. [41] explore the escape dynamics of bistable systems influenced by multiplicative noise. To this end, the authors use a generalized stochastic calculus framework to derive an analytical expression for the escape rate that is corroborated with numerical simulations. The results have applications in physics, chemistry and biology. The study of dynamical models continues with the article in [42], where Al-Saffar and Kim use a tumor-immune growth model to investigate how immunotherapy affects its dynamical characteristics. Their model is an extension of the prey–predator model of tumor cells and immune cells that includes periodic immunotherapy, nonlinear damping of cancer cells and the dynamics of a healthy cell population. The authors also study the effects of the model parameters.

As for the articles on physics, Monteoliva et al. [43] use information theory to investigate notable features of the quantum degree of mixedness (QDM) in a finite model of interacting fermions. The authors find that QDM exhibits a strong dependence on the total fermion number, with varying trends across different temperature regimes. Their results have potential implications for diverse fields ranging from condensed matter physics to quantum information science. In the context of non-collisional space plasmas, Consolini and De Michelis [44] explore irreversibility at ion and sub-ion scales during a magnetospheric current disruption event. Their results clearly demonstrate the irreversible nature of fluctuations at those scales. Hubay et al. [45] study turbulent flows over street canyons. The authors apply the quadrant method to transform time series of fluctuating velocities into symbolic sequences, enabling the investigation of their information content via word frequency and normalized entropy levels. Their results indicate that noisy periodic sequences exhibit entropy distributions qualitatively similar to those of the measured and simulated data.

Synchronization in nonlinear systems is a phenomenon of great interest. Lin and Pattanayak introduce in [46] the Difference Time Series Peak Complexity (DTSPC) algorithm, a technique that uses entropy as a tool to quantitatively measure synchronization. The authors show with coupled Lorenz systems that this technique reveals and quantifies the complexity of chaos synchronization, also capturing transitional behaviors between different regimes. Precisely, the article [47] contributed by Leyva et al. deals with transitions to synchronization, too, but of a particular kind: explosive transitions. Specifically, the authors propose using the ordinal pattern transition (OPT) entropy measured at sentinel central nodes as a potential predictor of explosive transitions to synchronization in networks of various dynamical systems. Their results show that the OPT entropic measure surpasses traditional early warning signal measures and could be a valuable tool for predicting critical transitions.

Among the many tools to study symbolic sequences, Hamming distance is tailored to the binary ones. Schneider and Zanette [48] study the structural properties of networks formed by random sets of bit strings; two bit strings are connected by a network link when they are sufficiently similar to each other, i.e., when their Hamming distance is below a certain threshold. Their results have to do with the degree distribution and the conditions for the existence of a giant component, as well as with clustering, assortativity and mean geodesic distance in this kind of network. Dec et al. [49] introduce a novel method of exploring linguistic networks by mapping word-adjacency networks to time series and applying multifractal analysis techniques. Their results indicate that the time series derived from clustering coefficients, when following the natural reading order, exhibits multifractal characteristics, revealing inherent complexity in textual organization. An analogous analysis based on the node degrees does not show such rich behaviors.

Finally, Sepúlveda and Amigó [50] provide a broad overview of a selection of generalized entropies and other entropic quantities, as well as their applications to data analysis and machine learning. This review also provides a brief historical account of the concept of entropy across several disciplines, its axiomatic formulation and a bibliometric survey of publications on this topic and their impact.

In conclusion, the above articles fittingly showcase the diversity of topics embraced by nonlinear dynamics, while also suggesting timely lines of research.
